# Supplementary material for: Transversal functional connectivity and scene-specific processing in the human entorhinal-hippocampal circuitry
Source: eLife. 2022 Oct 12;11:e76479. doi: 10.7554/eLife.76479 (PMC9651961; doi:10.7554/eLife.76479)
Supplement: Transparent reporting form [file elife-76479-transrepform1.pdf]

eLife

Westbrook Centre
Milton Road
Cambridge CB4 1YG, UK

P 01223 855340
W elifesciences.org
T @elife

## *eLife's* transparent reporting form

We encourage authors to provide detailed information *within their submission* to facilitate the interpretation and replication of experiments. Authors can upload supporting documentation to indicate the use of appropriate reporting guidelines for health-related research (see EQUATOR Network), life science research (see the BioSharing Information Resource), or the ARRIVE guidelines for reporting work involving animal research. Where applicable, authors should refer to any relevant reporting standards documents in this form.

If you have any questions, please consult our Journal Policies and/or contact us: editorial@elifesciences.org.

**Sample-size estimation**

- You should state whether an appropriate sample size was computed when the study was being designed
- You should state the statistical method of sample size computation and any required assumptions
- If no explicit power analysis was used, you should describe how you decided what sample (replicate) size (number) to use

Please outline where this information can be found within the submission (e.g., sections or figure legends), or explain why this information doesn't apply to your submission:

*We report results from 32 individuals and the data refers to the baseline measurement of a larger study. Our study advances Maass, Berron et al., 2015, eLIFE with similar analyses and our sample size is higher than the previous study's experiment-specific sample size (per experiment they used a sample size of around 20 individuals). We did not perform explicit power analyses but included all individuals that were qualifying for our study as it is quite difficult to get ultra-high resolution (f)MRI data that allows segmentation and analysis of individual hippocampal subfields. Please see participant information on page 22 (methods and participants).*

**Replicates**

- You should report how often each experiment was performed
- You should include a definition of biological versus technical replication
- The data obtained should be provided and sufficient information should be provided to indicate the number of independent biological and/or technical replicates
- If you encountered any outliers, you should describe how these were handled
- Criteria for exclusion/inclusion of data should be clearly stated
- High-throughput sequence data should be uploaded before submission, with a private link for reviewers provided (these are available from both GEO and ArrayExpress)

Please outline where this information can be found within the submission (e.g., sections or figure legends), or explain why this information doesn't apply to your submission:

eLife Sciences Publications, Ltd is a limited liability non-profit non-stock corporation incorporated in the State of Delaware, USA, with company number 5030732, and is registered in the UK with company number FC030576 and branch number BR015634 at the address Westbrook Centre, Milton Road, Cambridge CB4 1YG.

*All analyses were only performed once. They were performed in the way, we described in the manuscript (detailed analyses descriptions in page 23 to 28 of the manuscript and supplementary material page 13). None of the sampled 32 participants was excluded from an analysis.*

eLife Sciences Publications, Ltd is a limited liability non-profit non-stock corporation incorporated in the State of Delaware, USA, with company number 5030732, and is registered in the UK with company number FC030576 and branch number BR015634 at the address Westbrook Centre, Milton Road, Cambridge CB4 1YG.

**Statistical reporting**

- Statistical analysis methods should be described and justified
- Raw data should be presented in figures whenever informative to do so (typically when N per group is less than 10)
- For each experiment, you should identify the statistical tests used, exact values of N, definitions of center, methods of multiple test correction, and dispersion and precision measures (e.g., mean, median, SD, SEM, confidence intervals; and, for the major substantive results, a measure of effect size (e.g., Pearson's r, Cohen's d)
- Report exact p-values wherever possible alongside the summary statistics and 95% confidence intervals. These should be reported for all key questions and not only when the p-value is less than 0.05.

Please outline where this information can be found within the submission (e.g., sections or figure legends), or explain why this information doesn't apply to your submission:

*Statistical analyses methods and used statistical tests are precisely described in the in the methods section of the manuscript (data analysis page 23-28) and in the supplementary material (page 11-13). The sample size is described in the methods section. In the results section (page 5 – 12 and supplementary page 1 – 7, 11-12), relevant statistical values are reported along statistical tests. We report exact p-values. We present raw data whenever that is relevant (in figures 3, 4 and S3, S4, S9). Benjamini and Hochberg false-discovery-rate was used to correct for multiple comparison whenever that was relevant. This is stated in the methods (pages 23-28) and alongside the respective results.*

(For large datasets, or papers with a very large number of statistical tests, you may upload a single table file with tests, Ns, etc., with reference to sections in the manuscript.)

**Group allocation**

- Indicate how samples were allocated into experimental groups (in the case of clinical studies, please specify allocation to treatment method); if randomization was used, please also state if restricted randomization was applied
- Indicate if masking was used during group allocation, data collection and/or data analysis

Please outline where this information can be found within the submission (e.g., sections or figure legends), or explain why this information doesn't apply to your submission:

*We only analyzed data from one group of healthy individuals as described on page 22 (methods section, participants).*

**Additional data files ("source data")**

- We encourage you to upload relevant additional data files, such as numerical data that are represented as a graph in a figure, or as a summary table
- Where provided, these should be in the most useful format, and they can be uploaded as "Source data" files linked to a main figure or table
- Include model definition files including the full list of parameters used
- Include code used for data analysis (e.g., R, MatLab)
- Avoid stating that data files are "available upon request"

Please indicate the figures or tables for which source data files have been provided:

eLife Sciences Publications, Ltd is a limited liability non-profit non-stock corporation incorporated in the State of Delaware, USA, with company number 5030732, and is registered in the UK with company number FC030576 and branch number BR015634 at the address Westbrook Centre, Milton Road, Cambridge CB4 1YG.

Source data is provided under Grande, X., Berron, D. (2022). Open Science Framework. ID 9v3qp. Source Data from Functional Connectivity and Information Processing in the Entorhinal-Hippocampal Circuitry. https://osf.io/9v3qp

The Source Data for Figure 2 (SourceData_Figure2.mat) can be found in the tables T_rSub_fconn and T_rCA1_fconn. The columns within the ECseed columns refer to extracted connectivity values from each proximal to distal segment (1 – 5 or 1 – 3) of the respective region. Rows refer to individuals. The four EC seeds refer to the $EC_{RSC\text{-}based}$ seed (RSCECseed), $EC_{Area35\text{-}based}$ (A35ECseed), $EC_{PHC\text{-}based}$ (PHCECseed) and $EC_{Area36\text{-}based}$ (A36ECseed). Similarly, data for Appendix 1 Figure 2 (SourceData_Appendix1Figure2.mat) can be found in the tables T_lSub_fconn and T_lCA1_fconn.

Data for Figure 3 (SourceData_Figure3.mat) can be found in the table T_rEC_ObjectScene. The two columns within the seed columns refer to extracted parameter values for the object versus baseline and scene versus baseline conditions. Rows refer to individuals. The isthmuscingulate seed refers to the $EC_{RSC\text{-}based}$ seed; Area 35 to the $EC_{Area35\text{-}based}$; Area 36 to the $EC_{Area36\text{-}based}$ and PhC to the $EC_{PHC\text{-}based}$ seeds. Similarly, data for Appendix 1 - Figure 3 (SourceData_Appendix1Figure3.mat) can be found in the table T_lEC_ObjectScene.

Data for Figure 4 (SourceData_Figure4.mat) can be found in tables T_rSub_ObjectScene and T_rCA1_ObjectScene. The columns refer to extracted parameter values from each proximal to distal segment (1 – 5 or 1 – 3) of the respective region. Rows refer to individuals. The two conditions refer to the object versus baseline and the scene versus baseline parameter estimates. Similarly, data for Appendix 1 - Figure 4 (SourceData_Appendix1Figure4.mat) can be found in tables T_lSub_ObjectScene and T_lCA1_ObjectScene.

Data for Appendix 5 - Figure 1 (SourceData_Appendix5Figure1.mat) can be found in the table T_rSources_ObjectScene (right hemisphere) and T_rSources_ObjectScene (left hemisphere). The two columns within the seed columns refer to extracted parameter values for the object versus baseline and scene versus baseline conditions. Rows refer to individuals. The isthmuscingulate column refers to the RSC source; Area 35 to the Area 35 source region; Area 36 to Area 36 source and PHC to the PHC source region.

Group-level statistical maps (T statistics of one-samples T-tests, Source Code 1-8) from seed-to-voxel connectivity analysis between entorhinal cortices and retrosplenial cortex (ECseedResults_spmT_l_isthmuscingulate.nii.gz and ECseedResults_spmT_r_isthmuscingulate.nii.gz), parahippocampal cortex (ECseedResults_spmT_l_PhC.nii.gz and ECseedResults_spmT_r_PhC.nii.gz), Area 35 (ECseedResults_spmT_l_Area35.nii.gz and ECseedResults_spmT_r_Area35.nii.gz) and Area 36 (ECseedResults_spmT_l_Area36.nii.gz and ECseedResults_spmT_r_Area36.nii.gz), are stored for each seed and hemisphere separately and were used to generate the following figures: Figure 1; Appendix 1 – Figure 1; Appendix 3 – Figure 1; Appendix 4 – Figure 1

Group-level statistical maps (T statistics of one-samples T-tests, Source Code 9-16) from seed-to-voxel connectivity analysis between subiculum and CA1 and entorhinal cortex seeds $EC_{RSC\text{-}based}$ (HCResults_spmT_l_EC_isthmuscingulate.nii.gz and HCResults_spmT_r_EC_isthmuscingulate.nii.gz), $EC_{Area36\text{-}based}$ (HCResults_spmT_l_EC_Area36.nii.gz and HCResults_spmT_r_EC_Area36.nii.gz), $EC_{PHC\text{-}based}$ (HCResults_spmT_l_EC_PhC.nii.gz and HCResults_spmT_r_EC_PhC.nii.gz) and $EC_{Area35\text{-}based}$ (HCResults_spmT_l_EC_Area35.nii.gz and HCResults_spmT_r_EC_Area35.nii.gz) are stored for each seed and hemisphere separately and were used to generate the following figures: Figure 2; Appendix 1 – Figure 2; Appendix 3 – Figure 2; Appendix 4 – Figure 1

eLife Sciences Publications, Ltd is a limited liability non-profit non-stock corporation incorporated in the State of Delaware, USA, with company number 5030732, and is registered in the UK with company number FC030576 and branch number BR015634 at the address Westbrook Centre, Milton Road, Cambridge CB4 1YG.
